# Adipose tissue function in healthy pregnancy, gestational diabetes mellitus and pre-eclampsia

**DOI:** 10.1038/s41430-021-00948-9

**Published:** 2021-06-15

**Authors:** Cara Trivett, Zoe J. Lees, Dilys J. Freeman

**Affiliations:** Institute of Cardiovascular and Medical Sciences, University of Glasgow, Glasgow, UK

**Keywords:** Pathogenesis, Obesity

## Abstract

Gestational diabetes mellitus (GDM) is a common disorder of pregnancy with short- and long-term consequences for mother and baby. Pre-eclampsia is of major concern to obstetricians due to its sudden onset and increased morbidity and mortality for mother and baby. The incidence of these conditions continues to increase due to widespread maternal obesity. Maternal obesity is a risk factor for GDM and pre-eclampsia, yet our understanding of the role of adipose tissue and adipocyte biology in their aetiology is very limited. In this article, available data on adipose tissue and adipocyte function in healthy and obese pregnancy and how these are altered in GDM and pre-eclampsia are reviewed. Using our understanding of adipose tissue and adipocyte biology in non-pregnant populations, a role for underlying adipocyte dysfunction in the pathological pathways of these conditions is discussed.

## Introduction

Maternal obesity (body mass index [BMI] > 30 kg/m^2^) is a major public health concern which can predispose to higher rates of adverse pregnancy outcomes, particularly the metabolic disorders of gestational diabetes mellitus (GDM) and pre-eclampsia [1, 2]. GDM is defined as glucose intolerance of varying severity with first recognition during pregnancy and is characterised by β-cell dysfunction and exaggerated insulin resistance. Seventeen percent of live births globally are affected by hyperglycaemia and 85% of these have GDM [3]. GDM is associated with an increase in pregnancy complications including antenatal disease, prematurity, complicated delivery, postnatal complications, stillbirths, macrosomia, small for gestational age babies, congenital anomalies and neonatal mortality and morbidity. Pre-eclampsia, a multi-system disorder particular to human pregnancy is also a leading cause of maternal and neonatal morbidity and mortality. It is characterised by widespread endothelial dysfunction, resulting in hypertension due to vasoconstriction, proteinuria attributable to glomerular damage and oedema secondary to increased vascular permeability. Pre-eclampsia is a complex disease encompassing different phenotypic presentations (early/late onset, obesity-related or not, severe/mild) which may or may not share common aetiologies. The incidence of pre-eclampsia is 2–8% and 1.7% of pregnancies complicated by pre-eclampsia resulted in neonatal death, 4.6% in major neonatal morbidity and 67% in admission to a neonatal special care unit [4]. Women with pre-eclampsia have increased insulin resistance leading to a metabolic adaptation to pregnancy not dissimilar to metabolic syndrome [5]. This may explain at least in part the link with BMI, however, insulin resistance also predicts pre-eclampsia independent of maternal BMI [6].

As rates of maternal obesity have been increasing so has the incidence of these two diseases of pregnancy. The link with maternal obesity may be related to the adverse effects of adiposity on maternal metabolism and inflammation. However, meta-analysis of lifestyle interventions to reduce gestational weight gain (GWG) has shown that a reduction in GWG does not reduce adverse pregnancy outcomes including pre-eclampsia and GDM [7]. This suggests that the link between maternal obesity and risk of pre-eclampsia and GDM may not be straightforward. The aim of this review is to use our knowledge of adipose tissue function and how it contributes to metabolic disease in non-pregnant individuals to understand the pathological pathways which may underlie adiposity-related adverse pregnancy outcomes.

## Methodology

A systemised review of adipose tissue and adipocytes in GDM and pre-eclampsia was carried out. A search of published literature using PubMed and MEDLINE was conducted independently by two investigators using the keywords: “adipose” OR “adipocyte” OR “subcutaneous OR visceral OR omental OR ectopic NEAR fat” OR “lipolysis” combined with “pregnancy” OR “gestation” OR “maternal”, “gestational diabetes mellitus” OR “preeclampsia” OR “hypertens* NEAR pregnancy” using the operator AND. Duplicates were removed and all 2296 articles were screened by title and abstract for inclusion. Articles were excluded if: BMI was the only assessment of body fatness; there was a lack of healthy control group; an abstract could not be found or was a conference proceeding; only fetal outcomes were measured or the article was published in a language other than English. The number of total publications assessed was 176. This list was supplemented for the wider review with references that provided background information on non-pregnant adipose tissue function, healthy and obese adaptation to pregnancy and clinical features of healthy and complicated pregnancy. A maximum of 100 references could be cited in this article. Non-cited reference material is available as Supplementary References.

## Adipose tissue function in healthy non-pregnant individuals

Adipose tissue is not a single, uniform organ but is organised and distributed throughout the body into distinct anatomical and functional depots which determine its role in energy homeostasis and its association with disease. Adipose tissues are divided into three subtypes: white, brown and beige. Whilst brown adipose tissue has an important role in thermogenesis and beige adipose tissue is of emerging interest as a site of fat oxidation, white adipose tissue (WAT) is the primary site of lipid storage and mobilisation in humans and is the focus of this review. WAT acts as a buffer against the toxic effects of excess circulating lipids by storing free fatty acids (FFA) in adipocytes and releasing them for use by peripheral tissues in times of relative energy depletion. Peripheral, subcutaneous adipose tissue (SAT) is the largest depot and is the primary storage site of fatty acids as triglyceride. SAT can be located in the upper and lower body; lower body SAT is more insulin sensitive and stores fatty acids securely, whereas upper body SAT is relatively insulin resistant and has a greater propensity to release fatty acids from storage. Central, visceral adipose tissue (VAT) is associated with the development of obesity-related disorders, probably via the release of fatty acids directly into the portal circulation. The extent and site of lipid storage in response to energy excess is regulated by endocrine function, the rate of lipid utilisation and the expansion capacity of WAT.

Uptake of fatty acids is mediated via lipoprotein lipase (LPL) that is secreted by adipocytes and situated on the vascular endothelium of adipose tissue. LPL hydrolyses FFA from triglycerides contained in triglyceride-rich lipoproteins such as chylomicrons and Very low density lipoprotein (VLDL), allowing their uptake into adipocytes via fatty acid transporters in the adipocyte membrane. The release of FFA from adipose tissue is governed by lipolysis of triglyceride, regulated via a trio of enzymes in a stepwise manner: adipose triglyceride lipase, hormone sensitive lipase (HSL) and monoglyceride lipase. Insulin is a key hormone that inhibits adipocyte lipolysis and increases glucose uptake. Healthy adipose tissue displays metabolic flexibility, whereby lipolysis is carefully regulated by hormones, catecholamines and paracrine signals to facilitate physiological responses to changes in requirements for lipid storage versus mobilisation. FFA released from SAT are returned to the liver for oxidation or secretion in VLDL. Upper body SAT is estimated to be the source of 60% of circulating FFA, lower body SAT 15–20% and VAT 6–17% [8]. While VAT displays lower or equivalent rates of lipolysis to SAT, it has greater responsiveness to lipolytic stimuli and reduced responsiveness to inhibition by insulin.

Adipokines and cytokines secreted by adipocytes, adipose-resident immune cells, endothelial cells and other cellular compartments of the adipose tissue can have a diverse range of autocrine and paracrine effects which act to regulate insulin sensitivity, inflammation, cardiovascular function, behaviour and cell growth. Adiponectin, one of the most abundant adipokines in circulation, is anti-inflammatory, insulin sensitising and is inversely associated with increased adiposity. Leptin, the most well-characterised adipokine, correlates with fat mass and is increased post-prandially, regulating food intake and glucose and fat metabolism in healthy individuals. In contrast to adiponectin, leptin has pro-inflammatory actions and is associated with increased adiposity and insulin resistance. There are numerous other adipokines which are not the focus of this current review and are discussed elsewhere [9]. Obesity is a state of chronic low-grade inflammation and obesity-induced insulin resistance precedes macrophage accumulation and inflammation in adipose tissue [10]. Increased accumulation of immune cells and production of pro-inflammatory molecules is a key mechanism by which adipose tissue contributes to increasing insulin resistance and dyslipidaemia. Adipose tissue may convey signals to other tissues via the protein, lipid and microRNA content of extracellular vesicles produced and released by adipocytes or other cells within adipose tissue [11].

### Adipose tissue expandability, insulin resistance and cardio-metabolic disease

BMI is a universally accepted measure of body fatness and biomarker of metabolic disease risk when used in conjunction with other risk factors and is routinely measured in clinical settings. However, because BMI does not measure either the quantity or quality of body fat or convey any information about its location (SAT or VAT), it is relatively indiscriminate in predicting cardio-metabolic risk in comparison to central obesity with high intra-abdominal VAT. BMI is thus a proxy marker of adiposity, which cannot distinguish individuals who store fat in SAT in a relatively benign way, from those who store fat in VAT, which is related to metabolic dysfunction and pathology. In addition, the site of fat storage is recognised to be influenced by ethnicity, age, smoking and diet, which strongly suggests that the efficacy of BMI as a risk discriminator will vary between different populations.

Adipose tissue is comprised of adipocytes and stromal cells including pre-adipocytes and immune cells, which support the proliferation and differentiation of pre-adipocytes to adipocytes and secrete a variety of cytokines and growth factors facilitating this. When individuals become obese, excess calories are stored as triglyceride within the adipocytes of WAT. If there is insufficient capacity in mature adipocytes, new adipocytes are formed from pre-adipocytes in order to increase storage capacity. The formation of adipocytes (adipogenesis) occurs in two phases. The first phase is commitment to differentiation and involves the production of committed white pre-adipocytes from mesenchymal stem cells. Terminal differentiation forms mature adipocytes containing a lipid droplet that occupies almost all the space within the cell. In some individuals there appears to be a limited ability to produce mature adipocytes from pre-adipocytes (hyperplastic adipocyte expansion) and instead excess fatty acids are stored in existing mature adipocytes leading to an increase in their size (hypertrophic expansion) [12, 13]. In obese women hyperplasia is predominantly observed in SAT expansion, whereas hypertrophy is observed in both SAT and VAT adipocytes [14]. Larger adipocytes are dysfunctional and are demonstrably insulin resistant resulting in increased lipolysis due to resistance to the anti-lipolytic effects of insulin [15, 16]. Healthy individuals with a family history of type 2 diabetes mellitus (T2DM) show higher SAT diameter compared to BMI-matched controls and SAT size is independently correlated with insulin resistance in a variety of populations [17]. It is not clear to what degree adipocyte differentiation is limited by insulin resistance or whether insulin resistance results from impaired adipocyte differentiation or both. Failure of angiogenesis and the resulting inadequate blood supply to hypertrophic adipocytes leads to necrosis, macrophage infiltration into adipose tissue, inflammation and dysregulated adipokine release. Increased fibrosis is also associated with obese adipose tissue, further contributing to limited adipocyte expandability, tissue hypoxia and a pro-inflammatory environment.

Once SAT capacity is exceeded, lipids begin to accumulate in visceral and finally ectopic tissues [13]. “Spillover” of fatty acids unable to be retained in subcutaneous adipocytes, either due to an insufficient number of mature adipocytes or due to hypertrophic adipocytes becoming insulin resistant, leads to an increase in the visceral fat compartment and simultaneously a flux of fatty acids into ectopic sites, stored as intracellular lipid droplets in tissues such as liver, heart and pancreas. It is not the absolute amount of fat mass per se that determines adiposity-related risk, but it is the amount of VAT and ectopic fat accrued which is metabolically unfavourable [18]. The formation of ectopic fat is closely linked to the development of insulin resistance and T2DM. Thus, individuals with limited adipocyte expandability, such as South Asians, are at increased risk of T2DM [19]. In mouse models, inhibition of adipocyte lipolysis reduces both insulin resistance and ectopic fat accumulation [20]. Thus, adipocyte dysfunction plays an integral part in the pathological pathway of T2DM.

### Maternal metabolic adaption to pregnancy and gestational fat storage (Fig. 1A)

Adipose tissue expansion is an adaptive response to healthy human pregnancy. Maternal body composition and metabolism undergo dramatic changes in order to provide the developing fetus with a continuous supply of nutrients and prepare the mother for lactation. The initial phase of gestation is anabolic when maternal fat stores increase to a peak towards the end of the second trimester due to adipocyte hyperplasia and increased adipose tissue lipogenesis. Metabolism switches to a catabolic state around mid-gestation, characterised by increased adipose tissue fatty acid turnover and increased levels of lipolysis. Gluteal SAT adipocytes increased in size and number by late pregnancy in healthy non-obese women and adipose tissue showed increased expression of genes involved in tissue remodeling, angiogenesis and inflammation [21]. Increased rates of lipolysis are driven by maternal insulin resistance in response to gestational hormones, with adipocytes demonstrating reduced insulin suppression of lipolysis in late pregnancy compared to early pregnancy. Animal studies suggest there is a shift in the HSL to LPL expression ratio to favour lipolysis [22]. Lipolysis of maternal adipose stores accumulated in early pregnancy, coupled with reduced glucose uptake into maternal tissues, induced by maternal insulin resistance, ensures maximum nutrient availability for the developing fetus. In parallel with the accumulation of maternal adipose tissue, levels of circulating leptin in pregnancy increase to a maximum between 20 and 30 weeks of gestation. Low plasma levels of adiponectin are evident in pregnancy and appear to be linked to decreased insulin sensitivity of glucose disposal rather than lipid metabolism.

**Fig. 1:**
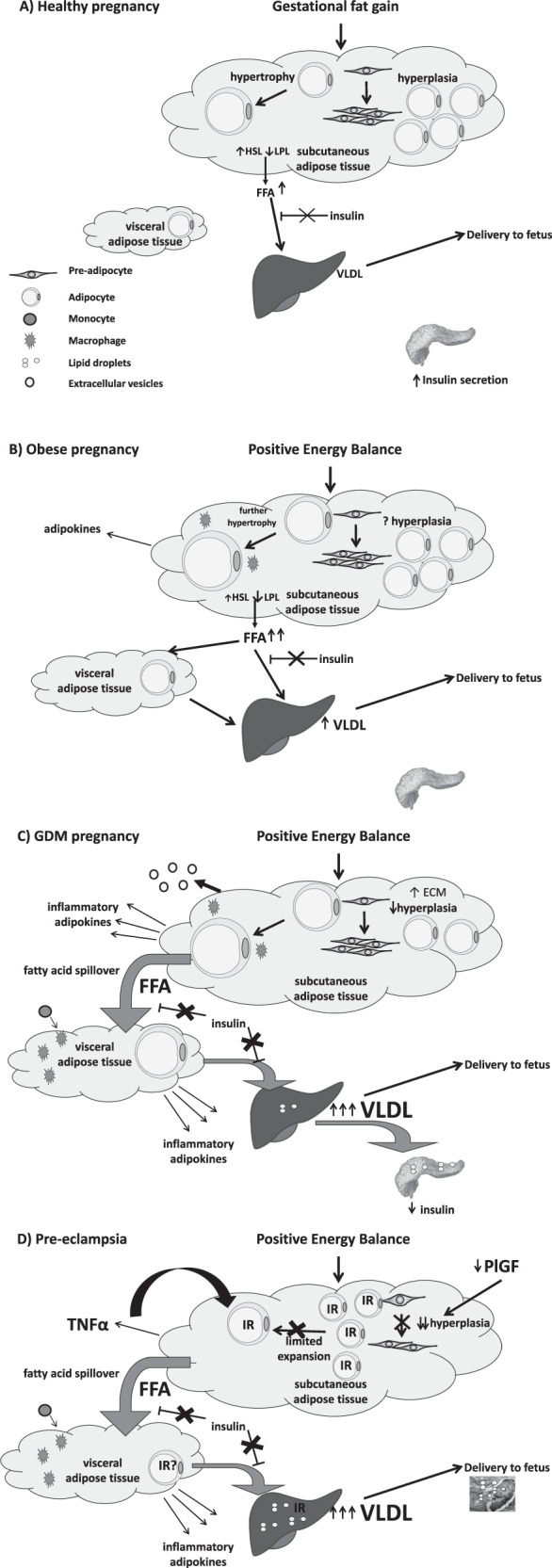
Adipocyte function in healthy, obese, GDM and pre-eclampsia pregnancy. **A** In healthy pregnancy gestational fat storage is accommodated by adipocyte hypertrophy. The physiologically healthy level of insulin resistance permits controlled adipocyte lipolysis in order to supply triglycerides to the fetus via placental uptake of fatty acids from maternal VLDL. **B** Obese women already exhibit SAT and VAT adipocyte hypertrophy prior to pregnancy. During pregnancy they develop a greater degree of insulin resistance than lean women which reduces the ability of insulin to downregulate adipocyte lipolysis. This results in increased FFA flux to the liver and increased liver VLDL production. **C** In women who develop GDM, it is hypothesised that there is reduced hyperplasia and SAT hypertrophy resulting in fatty acid spillover. These fatty acids can cascade to ectopic sites including VAT, the liver and the pancreas and may lead to pancreatic dysfunction β-cell failure and the development of GDM. **D** In women who develop pre-eclampsia, it is hypothesised that there is an innate maternal insulin resistance manifest in both SAT and VAT. This insulin resistance reduces adipocyte differentiation, reducing the number of mature adipocytes and thereby reducing the capacity of SAT to store fat. Fatty acid spillover occurs leading to hypertriglyceridaemia and deposition of ectopic fat in the liver and placenta contributing to further maternal insulin resistance and placental dysfunction. FFA free fatty acid, HSL hormone-sensitive lipase, LPL lipoprotein lipase, SAT subcutaneous adipose tissue, VAT visceral adipose tissue, VLDL very low density lipoprotein.

In pregnancy there is a shift in fat distribution from the lower body towards the upper abdominal region. The increase in abdominal fat in pregnancy has been found to be attributable to accumulation of VAT rather than SAT and there is an increase in VAT accumulation throughout gestation even in healthy pregnancy. Regional differences in SAT function were observed in pregnancy, with LPL activity in adipocytes from the femoral region, but not the abdominal region, increasing in pregnant women [23, 24]. Adiponectin mRNA expression does not differ between SAT and VAT in pregnant women [25].

### Maternal obesity and adipose tissue function (Fig. 1B)

Despite lean and obese women accumulating similar amounts of gestational fat, the distribution of adipose tissue storage varies dependent on pre-pregnancy BMI. Skinfold thickness measurements show that lean pregnant women gain significantly more fat in lower body compartments, whereas obese pregnant women gain more in upper body compartments [26]. Further, lean pregnant women gain weight at a quicker rate than overweight and obese pregnant women, and increased VAT accumulation is found in those who are overweight and obese [27]. VAT thickness in early pregnancy correlates better with metabolic risk factors than maternal BMI. VAT thickness in pregnancy has also been found to correlate with levels of C-reactive protein and glycated haemoglobin [28, 29]. An impaired immune response in pregnant women, indicated by decreased expression of CD4^+^ T cell cytokines, correlated with abdominal VAT but not SAT [30].

Consistent with non-pregnant women, obesity in pregnancy is characterised by insulin resistance, dyslipidaemia and low-grade chronic inflammation [31, 32]. SAT and VAT expression of genes involved in adipocyte fatty acid uptake were generally decreased in obese compared to lean women in the third trimester of pregnancy [33], while others such as fatty acid synthase were unchanged [33]. SAT and VAT expression of genes involved in adipocyte lipolysis and of transcription factors regulating fatty acid metabolism (LXRα, PPARα, PPARδ, RXRα, SREBP1c) and adipogenesis (PPARγ) were lower in obese compared to lean women [33]. Obese pregnant women were less able to adapt substrate metabolism in accordance with fuel availability, which was associated with increased postprandial inflammation and insulin resistance [31, 34].

The higher levels of inflammation in obese compared to lean women suggest that adipokines may play a role in the pathophysiology of obesity-related pregnancy, possibly via endothelial dysfunction [32]. There were higher levels of leptin in maternal obesity compared to lean pregnant women which contributed to leptin resistance [35]. Circulating levels of adiponectin were significantly lower in obese compared to lean pregnant women [31], however this was not associated with increased levels of pro-inflammatory cytokines [32, 36]. Hypoadiponectinemia in late obese pregnancy was attributable to reduced expression and methylation of the adiponectin gene [37]. When VAT tissue explants were studied ex vivo, VAT from obese women tended to secrete higher levels of pro-inflammatory mediators and upon exposure to inflammatory stimuli the tissue explants reduced the expression of genes involved in fatty acid uptake and lipolysis [33].

### Adipocyte function in lean and obese pregnancy

Adipocytes in abdominal SAT at gestational weeks 8–12 were non-significantly larger in obese pregnant women (mean diameter 91 µm) than lean pregnant women (79 µm) [38]. Obese women also had twice as many SAT adipocytes as lean women in the first trimester [38]. Between the first and third trimester, lean women significantly increased SAT adipocyte diameter (to 87 µm) but not adipocyte number, whereas obese women displayed no change in diameter (87 µm in the third trimester) or number over pregnancy [38]. There was evidence of a heterogeneous response where, when those who had gestational weight loss were excluded, obese women showed an increase in adipocyte number [38]. Third trimester SAT [39, 40] and VAT [41] adipocyte hypertrophy has also been reported in obese compared to lean third trimester pregnant women. Others have reported that obese women had fewer adipocytes and increased evidence of necrosis and apoptosis suggesting a higher adipocyte turnover and greater adipose tissue remodelling compared to lean pregnant women [39]. Analysis of the SAT and VAT transcriptome showed that a large number of genes in SAT had a gestational response compared to very few in VAT [42]. There was a trend towards lower SAT PPARγ expression in obese pregnant women compared to non-pregnant BMI-matched controls [43]. These data suggest that lean women undergo adipocyte hypertrophy in response to pregnancy, whereas obese women may already have adipocytes that have undergone hypertrophy prior to pregnancy. In obese women, gestational fat gain appears to be accommodated by an increase in cell number or increased adipose tissue remodelling. Some have observed no difference in SAT macrophage content between lean and obese women, and no gestational change in either group [38]. Others have identified higher SAT macrophage number [39] and a changed VAT macrophage population composition in maternal obesity [41].

Insulin binding to SAT adipocytes was reduced by more than 50% in the third trimester of pregnancy compared to non-pregnant controls [40, 44, 45] and was further decreased in obese pregnant women [40]. SAT adipocyte lipolytic activity increased over gestation in both lean and obese women despite obese women having a somewhat higher lipolytic activity in early pregnancy [38]. Insulin suppression of adipocyte lipolysis was decreased in obese compared to lean pregnant women [40]. Similarly, while there was no difference in adipocyte basal glucose uptake between non-pregnant and pregnant women, there was impaired insulin-stimulated glucose transport [44, 45] and evidence of defective post-receptor insulin signalling in pregnancy [44], although others disagree [45, 46]. Lower expression of GLUT1 and GLUT4 [47] and higher expression of IRS-2 were observed in adipocytes from obese pregnancies compared to obese non-pregnant adipose tissue [43] and in adipocytes from obese compared to non-obese pregnancies although this latter observation was not confirmed by protein expression [47]. There was also a trend towards lower basal and insulin-stimulated lipogenesis in pregnant women compared to non-pregnant [44]. IL-6 and TNFα release was not different between lean and obese groups and was unchanged over gestation, whereas adipocyte adiponectin release significantly declined in lean, but not obese, women. Adipocyte lipolysis was associated with insulin resistance assessed by Homeostasis Model Assessment (HOMA), in the first trimester and the proportion of large adipocytes correlated with insulin resistance in the third trimester [38].

### Adiposity and risk of metabolic disorders of pregnancy

**Fig. 2:**
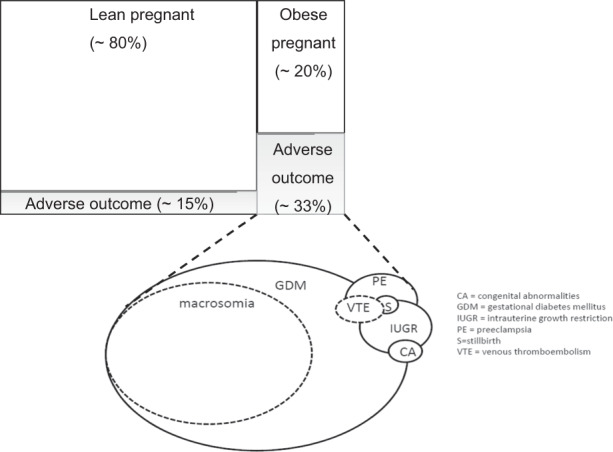
Body mass index is a poor discriminator of obesity-related adverse pregnancy outcome. Approximately 20% of all pregnancies are in mothers who are obese at the time of antenatal booking (around 11 weeks’ gestation) and undergo expensive, intensive antenatal monitoring. However, this percentage varies widely across different countries. Approximately one-third of obese mothers have an adverse pregnancy outcome. Obese mothers appear to be twice as likely to suffer an adverse pregnancy outcome than non-obese mothers, yet there is little information on how this varies between populations. There are a variety of obesity-related adverse outcomes of varying prevalence (represented by circle size). The underlying cause of adverse outcomes in obese mothers is often linked to metabolic dysregulation.

Pre-pregnancy BMI and early GWG are potentially modifiable risk factors for gestational hypertensive disorders and GDM [48–50]. Although BMI is a low-cost screening tool, the limitations of its effectiveness in discriminating between high- and low-risk pregnancy have not been formally tested. Importantly, only ~30% of obese pregnant women have an adverse pregnancy outcome [51]. Thus, 70 out of every 100 women identified as high risk undergo expensive, intensive antenatal monitoring unnecessarily (Fig. 2). Consistently GDM and PE populations can be reliably distinguished by measures of central fatness (SAT, VAT or total abdominal adipose tissue thickness) using ultrasonography and bioelectrical impedance. VAT thickness [52] and adiponectin, a metabolic biomarker associated with VAT [53], as well as waist circumference [54] strongly predict GDM. Similarly, prospective cohort studies for pre-eclampsia indicate measures of central/visceral obesity, i.e. waist circumference [55] and VAT thickness [56] are predictive. Thus far, large-scale multi-centre studies assessing the predictive power of measures of adipose tissue thickness relative to BMI are lacking and there is a lack of consensus on appropriate risk-defining cut-offs which may be influenced by ethnicity [19]. Recent studies also support SAT thickness as a predictor of GDM [57] although this may be acting as a proxy marker for visceral adiposity.

### Adipose tissue and adipocyte function in GDM (Fig. 1C)

Women with GDM had larger VAT adipocytes than normal glucose tolerant (NGT) controls and adipocyte size correlated with plasma glucose levels [58]. SAT expression of PPARγ was reduced in GDM compared to NGT controls [33, 43, 59] but others have found no differences in SAT or VAT PPARγ mRNA expression between GDM and NGT. Third trimester SAT and VAT expression of genes involved in adipocyte fatty acid uptake were decreased in GDM women compared to NGT women of similar BMI [33]. In addition, SAT and VAT expression of genes involved in triglyceride synthesis, fatty acid synthesis and triglyceride lipolysis were decreased in GDM pregnancies compared to NGT controls [33]. Others found alterations in expression of VAT fatty acid binding and storage proteins, such as perilipin 1, consistent with increased lipolysis [60]. Expression of transcription factors regulating fatty acid metabolism were lower in GDM women compared to NGT controls in SAT only [33].

Insulin receptor expression was found to be lower in both SAT and VAT from women with GDM compared to BMI-matched controls [61]. In comparative studies of non-obese and obese insulin-controlled GDM with BMI-matched controls, there was evidence for disruption in insulin receptor-signalling transduction pathway in SAT of GDM. Others have shown that defects in insulin-signalling pathways resided in VAT but not SAT. Neither SAT nor VAT leptin expression was different between GDM and BMI-matched NGT controls [62]. Many mediators have been proposed to induce insulin resistance in adipocytes in GDM. Plasma levels of an inhibitor of LPL, angiopoietin 4 (ANGPTL4), are decreased in GDM pregnancy [63]. In a mouse adipocyte cell model (3T3-L1), downregulation of ANGPTL4 expression inhibited adipocyte glucose uptake and insulin signalling and increased expression of pro-inflammatory cytokines [63]. VAT adipocyte plasma membrane-associated protein (APMAP) was found to be downregulated in GDM [64]. In the 3T3-L1 model, inhibition of APMAP expression resulted in impaired insulin signalling and activated NFκB-mediated inflammatory signalling [64]. A higher number of extracellular vesicles of different proteomic composition were produced by VAT explants from GDM compared to NGT women [65]. The extracellular vesicles from the GDM explants increased the expression of genes involved in glucose metabolism in placental cells [65].

In a cohort matched for BMI, VAT, but not SAT, TNFα mRNA expression was higher in GDM compared to controls, whereas IL-6 expression was unchanged in both tissues [66]. Leptin secretion from SAT explants was higher in GDM compared to NGT controls in both insulin-controlled and diet-controlled GDM [67] but unaffected by exposure to a variety of cytokines and hormones. SAT adiponectin expression was lower in VAT in GDM compared to BMI-matched controls [66]. Proteomic analyses showed in adipose tissue from GDM women that there was increased expression of inflammatory proteins and differential expression of proteins involved in mitochondrial dysfunction, sirtuin signalling and oxidative phosphorylation compared to BMI-matched NGT controls [60]. VAT, but not SAT, from women with GDM had higher macrophage infiltration compared to NGT controls and the degree of infiltration correlated with systemic insulin resistance, however these groups were not BMI-matched [68]. VAT tissue explants from GDM compared to NGT secreted higher levels of leptin but not IL-1β, or TNFα and others have found increased VAT leptin expression in GDM [69]. Expression of genes that co-ordinate pro- and anti-inflammatory pathways have been found to be altered in VAT from GDM [70]. Apart from a higher IL-8 expression in VAT, there was no difference in SAT and VAT mRNA expression of pro-inflammatory IL-6, IL-6 receptor and suppressor of cytokine signalling proteins 1 and 3 between GDM and BMI-matched NGT controls [71]. VAT from women with GDM showed higher expression and secretion of angiogenic proteins such as fms-like tyrosine kinase-1 (sflt-1), fibroblast growth factor (FGF2) and endoglin than BMI-matched controls [72]. Proteomics also revealed a higher expression of proteins involved in extracellular matrix in GDM VAT, suggesting a less flexible tissue which may limit expansion [60].

### Adipose tissue and adipocyte function in pre-eclampsia (Fig. 1D)

Published data on adipocyte function in pre-eclampsia is extremely limited. The size distribution of SAT and VAT adipocytes was altered in pre-eclampsia with more adipocytes in the lowest tertile of diameters compared to controls suggesting limited adipocyte differentiation [73]. Women with pre-eclampsia show systemic insulin resistance [74] and increased whole body lipolysis [75]. In pre-eclampsia, mature adipocytes were functionally insulin resistant as evidenced by a reduced suppression by insulin of β-adrenergic stimulated lipolysis [73]. Women with pre-eclampsia have higher plasma levels of triglycerides when compared to healthy BMI-matched controls [76] which is evident before clinical manifestation of pre-eclampsia [77] and suggests there may be increased rates of adipocyte lipolysis in women who go on to develop pre-eclampsia. Studies in placental growth factor (PlGF)-deficient mice suggest that reduced PlGF, as is observed in pre-eclampsia in humans, is associated with impaired adipose tissue development and vascularisation and the development of insulin resistance [78]. Both SAT and VAT adiponectin mRNA expression were unaltered between control and pre-eclampsia BMI-matched mothers [25].

In VAT, there was increased activated macrophage infiltration in pre-eclampsia and ex vivo adipocytes isolated from women with pre-eclampsia had higher inflammatory cytokine release in response to a pro-inflammatory stimulus [79]. Neutrophil infiltration was observed in the resistance vessels of SAT of women with pre-eclampsia [80]. Exposure of primary human adipocytes to human serum from women with pre-eclampsia increased mRNA expression of IL-6 and MCP-1 when compared to exposure to control serum [81]. In contrast, transcriptome profiling data from non-pregnant VAT exposed to pre-eclampsia sera suggested that these non-pregnant adipocytes are capable of mounting an anti-inflammatory response to the sera [82]. It was suggested that in vivo adipocyte inflammation might be provoked by advanced glycation end products or lipopolysaccharide [81]. Interestingly, despite pre-eclampsia being a disease of endothelial activation, there was no evidence of increased expression of pro-inflammatory endothelial activation markers in SAT from women with pre-eclampsia [83]. However, others have found increased expression of TNFα and ICAM-1 in SAT from women with pre-eclampsia compared to controls [84]. Increased VAT adipocyte expression of TNFα in pre-eclampsia has been observed and VAT adipocyte TNFα release was inversely associated with VAT adipocyte insulin sensitivity [79].

### Evidence for ectopic fat accumulation in healthy and complicated pregnancy

As described above, a failure of adipocyte expansion can lead to ectopic fat accumulation and development of insulin resistance and metabolic disease [18]. There is no evidence of ectopic fat accumulation in healthy pregnancy [85]. There is however evidence for this pathological pathway in pregnancies complicated by GDM and pre-eclampsia. Neither liver nor intra-myocellular fat was detected in women with GDM using magnetic resonance spectroscopy [86]. Plasma levels of gamma-glutaryl transferase, an indirect biochemical marker of liver fat, were predictive of GDM [87]. Recently, the presence of non-alcoholic fatty liver disease (NAFLD) during the initial period of gestation was identified as an independent risk factor for development of GDM [88]. In women with a history of GDM, liver fat rather than body composition was associated with insulin resistance [89], and these women develop NAFLD later in life [90]. There is evidence for ectopic fat accumulation in pre-eclampsia. Classically, the disease is characterised by atherosis of spiral arteries and the accumulation of lipids within glomerular endothelial cells [91]. This process is even more marked in the haemolysis, elevated liver enzymes and low platelet count syndrome and acute fatty liver of pregnancy, which are severe complications related to pre-eclampsia. Elevated plasma triglycerides were observed in pre-eclampsia [92], which is indicative of ectopic fat accumulation [93]. There is further indirect evidence of ectopic liver fat in pre-eclampsia as reduced long chain polyunsaturated fatty acid synthesis was observed in mothers with pre-eclampsia [94]. Placenta in pre-eclampsia had a higher total and neutral lipid (triglyceride and cholesteryl ester) content than in BMI-matched controls, which is preliminary evidence suggesting that ectopic lipid droplets may form in the placenta [95]. In addition, epicardial adipose thickness, an ectopic site of fat accumulation and a marker of visceral fat, was shown to predict both PE and GDM [96–98].

## Summary (Fig. 1)

Impairment of adipocyte expansion is related to development of T2DM in non-pregnant populations and an understanding of this pathway sheds light on adipocyte function in pregnancy. GWG can easily be accommodated by adipocyte hypertrophy in healthy pregnancy. The relatively limited insulin resistance induced by pregnancy hormones in health leads to a regulated increase in lipolysis to supply nutrients to the fetus. In obese pregnancy, women commence pregnancy with a relative degree of SAT and VAT adipocyte hypertrophy and insulin resistance. In response to pregnancy hormones, obese women develop a higher than normal insulin resistance which reduces the ability of insulin to switch off lipolysis resulting in increased delivery of fatty acids to VAT and the liver leading to increased liver VLDL production and increased plasma triglyceride concentrations. There are limited data on adipocyte function in GDM and pre-eclampsia but that which is available suggests a role for adipocyte dysfunction in these diseases of pregnancy. In women who develop GDM, one might hypothesise that there is reduced hyperplasia and SAT hypertrophy resulting in fatty acid spillover. These fatty acids are directed to VAT which also experiences adipocyte hypertrophy and a reduced ability to retain fatty acids. Once the capacity of both depots is exceeded, then ectopic fat may begin to collect in the liver and pancreas leading to reduced pancreatic insulin secretion and tipping maternal metabolism into overt GDM. In women with pre-eclampsia, one might hypothesise that there is an innate maternal insulin resistance perhaps induced by changes in placental hormones secreted by a dysfunctional placenta. This insulin resistance impairs adipocyte differentiation, limiting the number of mature adipocytes and hence severely limiting the capacity of SAT to store gestationally acquired fat. Fatty acid spillover will occur, resulting in increased plasma lipids and ectopic fat accumulation in liver and placenta exacerbating further maternal insulin resistance and placental dysfunction. Clearly the evidence to support these hypotheses is limited and more data that will provide an understanding of the function of adipose tissue in GDM and pre-eclampsia is required.

## Future perspectives

Metabolic complications of obese pregnancy may share a common metabolic origin with those found in non-pregnant obesity, through the development of insulin resistance. Although the concepts of benign and visceral obesity are only just beginning to be addressed in obstetric medicine, recognition of these obese phenotypes could have a major impact on the ability to identify high risk pregnancy. Currently, the ability to predict the onset of gestational disorders is limited, despite robust associations with BMI. A more detailed understanding of the pathological pathways related to adiposity may identify new risk factors to predict, and potential new treatments to prevent, GDM and pre-eclampsia.

## Supplementary information

Supplemental References
