# Supplementary material for: Adipose tissue function in healthy pregnancy, gestational diabetes mellitus and pre-eclampsia
Source: Eur J Clin Nutr. 2021 Jun 15;75(12):1745–56. doi: 10.1038/s41430-021-00948-9 (PMC8636251; doi:10.1038/s41430-021-00948-9)
Supplement: Supplementary file 1 — Supplemental References [file 41430_2021_948_MOESM1_ESM.docx]

**Adipose tissue function in healthy pregnancy, gestational diabetes mellitus and pre-eclampsia**

**Cara Trivett, Zoe J. Lees, Dilys J. Freeman**

Institute of Cardiovascular and Medical Sciences, University of Glasgow, Glasgow, G12 8QQ

**Supplemental References**

1. Jarvie E, Ramsay JE. Obstetric management of obesity in pregnancy. *Seminars in fetal & neonatal medicine* 2010; **15**(2)**:** 83-88. e-pub ahead of print 2009/11/03; doi: 10.1016/j.siny.2009.10.001

2. Hod M, Pretty M, Mahmood T. Joint position statement on universal screening for GDM in Europe by FIGO, EBCOG and EAPM. *Eur J Obstet Gynecol Reprod Biol* 2018; **228:** 329-330. e-pub ahead of print 2018/06/14; doi: 10.1016/j.ejogrb.2018.05.037

3. Kapur A, Mahmood T, Hod M. The unmet need for universal testing for hyperglycaemia in pregnancy and the FIGO guideline. *BJOG : an international journal of obstetrics and gynaecology* 2018; **125**(5)**:** 529-531. e-pub ahead of print 2017/04/04; doi: 10.1111/1471-0528.14659

4. Clark P, Sattar N, Walker ID, Greer IA. The Glasgow Outcome, APCR and Lipid (GOAL) Pregnancy Study: significance of pregnancy associated activated protein C resistance. *Thrombosis and haemostasis* 2001; **85**(1)**:** 30-35. e-pub ahead of print 2001/02/24;

5. *Maternal obesity in the UK: Findings from a national project.* Centre for Maternal and Child Enquiries (CMACE). London, 2010.

6. Fingar KR, Mabry-Hernandez I, Ngo-Metzger Q, Wolff T, Steiner CA, Elixhauser A. Delivery Hospitalizations Involving Preeclampsia and Eclampsia, 2005–2014: Statistical Brief #222. In: *Healthcare Cost and Utilization Project (HCUP) Statistical Briefs*. Agency for Healthcare Research and Quality (US): Rockville (MD), 2006.

7. Data on Selected Pregnancy Complications in the United States. In: Centers for Disease Control and Prevention, 2019.

8. Ferrara A. Increasing prevalence of gestational diabetes mellitus: a public health perspective. *Diabetes care* 2007; **30 Suppl 2:** S141-146. e-pub ahead of print 2007/07/13; doi: 10.2337/dc07-s206

9. Lavery JA, Friedman AM, Keyes KM, Wright JD, Ananth CV. Gestational diabetes in the United States: temporal changes in prevalence rates between 1979 and 2010. *BJOG : an international journal of obstetrics and gynaecology* 2017; **124**(5)**:** 804-813. e-pub ahead of print 2016/08/12; doi: 10.1111/1471-0528.14236

10. Huda SS SN, Freeman DJ. Lipoprotein metabolism and vascular complications in pregnancy. *Clinical Lipidology* 2009; **4**(1)**:** 91-102.

11. Iacobini C, Pugliese G, Blasetti Fantauzzi C, Federici M, Menini S. Metabolically healthy versus metabolically unhealthy obesity. *Metabolism: clinical and experimental* 2019; **92:** 51-60. e-pub ahead of print 2018/11/21; doi: 10.1016/j.metabol.2018.11.009

12. Spiegelman BM. Banting Lecture 2012: Regulation of adipogenesis: toward new therapeutics for metabolic disease. *Diabetes* 2013; **62**(6)**:** 1774-1782. e-pub ahead of print 2013/05/25; doi: 10.2337/db12-1665

13. Tam CS, Lecoultre V, Ravussin E. Brown adipose tissue: mechanisms and potential therapeutic targets. *Circulation* 2012; **125**(22)**:** 2782-2791. e-pub ahead of print 2012/06/06; doi: 10.1161/circulationaha.111.042929

14. Goodpaster BH, Sparks LM. Metabolic Flexibility in Health and Disease. *Cell Metab* 2017; **25**(5)**:** 1027-1036. e-pub ahead of print 2017/05/04; doi: 10.1016/j.cmet.2017.04.015

15. Klein S. The case of visceral fat: argument for the defense. *The Journal of clinical investigation* 2004; **113**(11)**:** 1530-1532. e-pub ahead of print 2004/06/03; doi: 10.1172/jci22028

16. Nielsen TS, Jessen N, Jørgensen JO, Møller N, Lund S. Dissecting adipose tissue lipolysis: molecular regulation and implications for metabolic disease. *J Mol Endocrinol* 2014; **52**(3)**:** R199-222. e-pub ahead of print 2014/03/01; doi: 10.1530/jme-13-0277

17. Smith U, Kahn BB. Adipose tissue regulates insulin sensitivity: role of adipogenesis, de novo lipogenesis and novel lipids. *J Intern Med* 2016; **280**(5)**:** 465-475. e-pub ahead of print 2016/10/21; doi: 10.1111/joim.12540

18. Luo L, Liu M. Adipose tissue in control of metabolism. *J Endocrinol* 2016; **231**(3)**:** R77-r99. e-pub ahead of print 2016/12/10; doi: 10.1530/joe-16-0211

19. Kojta I, Chacińska M, Błachnio-Zabielska A. Obesity, Bioactive Lipids, and Adipose Tissue Inflammation in Insulin Resistance. *Nutrients* 2020; **12**(5). e-pub ahead of print 2020/05/08; doi: 10.3390/nu12051305

20. Svensson H, Odén B, Edén S, Lönn M. Adiponectin, chemerin, cytokines, and dipeptidyl peptidase 4 are released from human adipose tissue in a depot-dependent manner: an in vitro system including human serum albumin. *BMC Endocr Disord* 2014; **14:** 7. e-pub ahead of print 2014/01/23; doi: 10.1186/1472-6823-14-7

21. López-Jaramillo P, Gómez-Arbeláez D, López-López J, López-López C, Martínez-Ortega J, Gómez-Rodríguez A *et al.* The role of leptin/adiponectin ratio in metabolic syndrome and diabetes. *Hormone molecular biology and clinical investigation* 2014; **18**(1)**:** 37-45. e-pub ahead of print 2014/11/13; doi: 10.1515/hmbci-2013-0053

22. Lee MJ, Wu Y, Fried SK. Adipose tissue heterogeneity: implication of depot differences in adipose tissue for obesity complications. *Mol Aspects Med* 2013; **34**(1)**:** 1-11. e-pub ahead of print 2012/10/17; doi: 10.1016/j.mam.2012.10.001

23. de Heredia FP, Gomez-Martinez S, Marcos A. Obesity, inflammation and the immune system. *The Proceedings of the Nutrition Society* 2012; **71**(2)**:** 332-338. e-pub ahead of print 2012/03/21; doi: S0029665112000092 [pii]10.1017/S0029665112000092 [doi]

24. Neeland IJ, Ayers CR, Rohatgi AK, Turer AT, Berry JD, Das SR *et al.* Associations of visceral and abdominal subcutaneous adipose tissue with markers of cardiac and metabolic risk in obese adults. *Obesity (Silver Spring, Md.)* 2013; **21**(9)**:** E439-447. e-pub ahead of print 2013/05/21; doi: 10.1002/oby.20135

25. Stefan N, Kantartzis K, Machann J, Schick F, Thamer C, Rittig K *et al.* Identification and characterization of metabolically benign obesity in humans. *Archives of internal medicine* 2008; **168**(15)**:** 1609-1616. e-pub ahead of print 2008/08/13; doi: 10.1001/archinte.168.15.1609

26. Taylor AE, Ebrahim S, Ben-Shlomo Y, Martin RM, Whincup PH, Yarnell JW *et al.* Comparison of the associations of body mass index and measures of central adiposity and fat mass with coronary heart disease, diabetes, and all-cause mortality: a study using data from 4 UK cohorts. *The American journal of clinical nutrition* 2010; **91**(3)**:** 547-556. e-pub ahead of print 2010/01/22; doi: 10.3945/ajcn.2009.28757

27. Wannamethee SG, Papacosta O, Whincup PH, Carson C, Thomas MC, Lawlor DA *et al.* Assessing prediction of diabetes in older adults using different adiposity measures: a 7 year prospective study in 6,923 older men and women. *Diabetologia* 2010; **53**(5)**:** 890-898. doi: 10.1007/s00125-010-1670-7

28. Gimble JM, Bunnell BA, Frazier T, Rowan B, Shah F, Thomas-Porch C *et al.* Adipose-derived stromal/stem cells: a primer. *Organogenesis* 2013; **9**(1)**:** 3-10. e-pub ahead of print 2013/03/30; doi: 10.4161/org.24279

29. Arner P, Spalding KL. Fat cell turnover in humans. *Biochem Biophys Res Commun* 2010; **396**(1)**:** 101-104. e-pub ahead of print 2010/05/25; doi: 10.1016/j.bbrc.2010.02.165

30. Cristancho AG, Lazar MA. Forming functional fat: a growing understanding of adipocyte differentiation. *Nature reviews. Molecular cell biology* 2011; **12**(11)**:** 722-734. e-pub ahead of print 2011/09/29; doi: 10.1038/nrm3198

31. Elbers JM, de Jong S, Teerlink T, Asscheman H, Seidell JC, Gooren LJ. Changes in fat cell size and in vitro lipolytic activity of abdominal and gluteal adipocytes after a one-year cross-sex hormone administration in transsexuals. *Metabolism: clinical and experimental* 1999; **48**(11)**:** 1371-1377. e-pub ahead of print 1999/12/03;

32. Arner E, Ryden M, Arner P. Tumor necrosis factor alpha and regulation of adipose tissue. *The New England journal of medicine* 2010; **362**(12)**:** 1151-1153. e-pub ahead of print 2010/03/26; doi: 10.1056/NEJMc0910718

33. Accili D, Taylor SI. Targeted inactivation of the insulin receptor gene in mouse 3T3-L1 fibroblasts via homologous recombination. *Proc Natl Acad Sci U S A* 1991; **88**(11)**:** 4708-4712. e-pub ahead of print 1991/06/01; doi: 10.1073/pnas.88.11.4708

34. Engin A. Adipose Tissue Hypoxia in Obesity and Its Impact on Preadipocytes and Macrophages: Hypoxia Hypothesis. *Adv Exp Med Biol* 2017; **960:** 305-326. e-pub ahead of print 2017/06/07; doi: 10.1007/978-3-319-48382-5_13

35. Henninger AM, Eliasson B, Jenndahl LE, Hammarstedt A. Adipocyte hypertrophy, inflammation and fibrosis characterize subcutaneous adipose tissue of healthy, non-obese subjects predisposed to type 2 diabetes. *PLoS One* 2014; **9**(8)**:** e105262. e-pub ahead of print 2014/08/26; doi: 10.1371/journal.pone.0105262

36. Halberg N, Khan T, Trujillo ME, Wernstedt-Asterholm I, Attie AD, Sherwani S *et al.* Hypoxia-inducible factor 1alpha induces fibrosis and insulin resistance in white adipose tissue. *Molecular and cellular biology* 2009; **29**(16)**:** 4467-4483. e-pub ahead of print 2009/06/24; doi: 10.1128/mcb.00192-09

37. Abeysekera MV, Morris JA, Davis GK, O'Sullivan AJ. Alterations in energy homeostasis to favour adipose tissue gain: A longitudinal study in healthy pregnant women. *Aust N Z J Obstet Gynaecol* 2016; **56**(1)**:** 42-48. e-pub ahead of print 2016/01/29; doi: 10.1111/ajo.12398

38. Pipe NG, Smith T, Halliday D, Edmonds CJ, Williams C, Coltart TM. Changes in fat, fat-free mass and body water in human normal pregnancy. *British journal of obstetrics and gynaecology* 1979; **86**(12)**:** 929-940. e-pub ahead of print 1979/12/01; doi: 10.1111/j.1471-0528.1979.tb11240.x

39. Sidebottom AC, Brown JE, Jacobs DR, Jr. Pregnancy-related changes in body fat. *Eur J Obstet Gynecol Reprod Biol* 2001; **94**(2)**:** 216-223. e-pub ahead of print 2001/02/13; doi: 10.1016/s0301-2115(00)00329-8

40. Andersen O, Kühl C. Adipocyte insulin receptor binding and lipogenesis at term in normal pregnancy. *European journal of clinical investigation* 1988; **18**(6)**:** 575-581. e-pub ahead of print 1988/12/01; doi: 10.1111/j.1365-2362.1988.tb01270.x

41. Diderholm B, Stridsberg M, Ewald U, Lindeberg-Norden S, Gustafsson J. Increased lipolysis in non-obese pregnant women studied in the third trimester. *BJOG : an international journal of obstetrics and gynaecology* 2005; **112**(6)**:** 713-718. e-pub ahead of print 2005/06/01; doi: 10.1111/j.1471-0528.2004.00534.x

42. Elliott JA. The effect of pregnancy on the control of lipolysis in fat cells isolated from human adipose tissue. *European journal of clinical investigation* 1975; **5**(2)**:** 159-163. e-pub ahead of print 1975/04/01; doi: 10.1111/j.1365-2362.1975.tb00442.x

43. Catalano PM, Tyzbir ED, Roman NM, Amini SB, Sims EA. Longitudinal changes in insulin release and insulin resistance in nonobese pregnant women. *American journal of obstetrics and gynecology* 1991; **165**(6 Pt 1)**:** 1667-1672. e-pub ahead of print 1991/12/01;

44. Sivan E, Homko CJ, Chen X, Reece EA, Boden G. Effect of insulin on fat metabolism during and after normal pregnancy. *Diabetes* 1999; **48**(4)**:** 834-838. e-pub ahead of print 1999/04/02; doi: 10.2337/diabetes.48.4.834

45. Hodson K, Man CD, Smith FE, Thelwall PE, Cobelli C, Robson SC *et al.* Mechanism of insulin resistance in normal pregnancy. *Horm Metab Res* 2013; **45**(8)**:** 567-571. e-pub ahead of print 2013/04/04; doi: 10.1055/s-0033-1337988

46. Sattar N, Greer IA, Pirwani I, Gibson J, Wallace AM. Leptin levels in pregnancy: marker for fat accumulation and mobilization? *Acta Obstet Gynecol Scand* 1998; **77**(3)**:** 278-283. e-pub ahead of print 1998/04/16;

47. Sivan E, Whittaker PG, Sinha D, Homko CJ, Lin M, Reece EA *et al.* Leptin in human pregnancy: the relationship with gestational hormones. *American journal of obstetrics and gynecology* 1998; **179**(5)**:** 1128-1132. e-pub ahead of print 1998/11/20; doi: 10.1016/s0002-9378(98)70118-8

48. Catalano PM, Hoegh M, Minium J, Huston-Presley L, Bernard S, Kalhan S *et al.* Adiponectin in human pregnancy: implications for regulation of glucose and lipid metabolism. *Diabetologia* 2006; **49**(7)**:** 1677-1685. e-pub ahead of print 2006/06/06; doi: 10.1007/s00125-006-0264-x

49. Morgan SA, Bringolf JB, Seidel ER. Visfatin expression is elevated in normal human pregnancy. *Peptides* 2008; **29**(8)**:** 1382-1389. e-pub ahead of print 2008/06/06; doi: 10.1016/j.peptides.2008.04.010

50. Kasher-Meron M, Mazaki-Tovi S, Barhod E, Hemi R, Haas J, Gat I *et al.* Chemerin concentrations in maternal and fetal compartments: implications for metabolic adaptations to normal human pregnancy. *J Perinat Med* 2014; **42**(3)**:** 371-378. e-pub ahead of print 2013/12/18; doi: 10.1515/jpm-2013-0166

51. Aktas G, Alcelik A, Ozlu T, Tosun M, Tekce BK, Savli H *et al.* Association between omentin levels and insulin resistance in pregnancy. *Exp Clin Endocrinol Diabetes* 2014; **122**(3)**:** 163-166. e-pub ahead of print 2014/03/20; doi: 10.1055/s-0034-1370917

52. Stevens-Simon C, Thureen P, Barrett J, Stamm E. Skinfold caliper and ultrasound assessments of change in the distribution of subcutaneous fat during adolescent pregnancy. *International journal of obesity and related metabolic disorders : journal of the International Association for the Study of Obesity* 2001; **25**(9)**:** 1340-1345. e-pub ahead of print 2001/09/26; doi: 10.1038/sj.ijo.0801685

53. Selovic A, Sarac J, Missoni S. Changes in adipose tissue distribution during pregnancy estimated by ultrasonography. *The journal of maternal-fetal & neonatal medicine : the official journal of the European Association of Perinatal Medicine, the Federation of Asia and Oceania Perinatal Societies, the International Society of Perinatal Obstet* 2016; **29**(13)**:** 2131-2137. e-pub ahead of print 2015/09/15; doi: 10.3109/14767058.2015.1077220

54. Kinoshita T, Itoh M. Longitudinal variance of fat mass deposition during pregnancy evaluated by ultrasonography: the ratio of visceral fat to subcutaneous fat in the abdomen. *Gynecologic and obstetric investigation* 2006; **61**(2)**:** 115-118. e-pub ahead of print 2005/11/08; doi: 10.1159/000089456

55. Maple-Brown LJ, Roman NM, Thomas A, Presley LH, Catalano PM. Perinatal factors relating to changes in maternal body fat in late gestation. *Journal of perinatology : official journal of the California Perinatal Association* 2013; **33**(12)**:** 934-938. e-pub ahead of print 2013/09/14; doi: 10.1038/jp.2013.109

56. Ahmadi F, Moukhah S, Hosseini R, Maghari A. Ultrasound Evaluation of Visceral Fat Thickness for Prediction of Metabolic Syndrome in the First Trimester of Pregnancy in a Sample of Non-obese Iranian Women. *Oman Med J* 2019; **34**(4)**:** 308-312. e-pub ahead of print 2019/07/31; doi: 10.5001/omj.2019.61

57. Bartha JL, Marín-Segura P, González-González NL, Wagner F, Aguilar-Diosdado M, Hervias-Vivancos B. Ultrasound evaluation of visceral fat and metabolic risk factors during early pregnancy. *Obesity (Silver Spring, Md.)* 2007; **15**(9)**:** 2233-2239. e-pub ahead of print 2007/09/25; doi: 10.1038/oby.2007.265

58. De Souza LR, Kogan E, Berger H, Alves JG, Lebovic G, Retnakaran R *et al.* Abdominal adiposity and insulin resistance in early pregnancy. *J Obstet Gynaecol Can* 2014; **36**(11)**:** 969-975. e-pub ahead of print 2015/01/13; doi: 10.1016/s1701-2163(15)30409-6

59. Fattah C, Barry S, O'Connor N, Farah N, Stuart B, Turner MJ. Maternal leptin and body composition in the first trimester of pregnancy. *Gynecol Endocrinol* 2011; **27**(4)**:** 263-266. e-pub ahead of print 2010/06/10; doi: 10.3109/09513590.2010.491167

60. Jarvie EM, Stewart FM, Ramsay JE, Brown EA, Meyer BJ, Olivecrona G *et al.* Maternal Adipose Tissue Expansion, A Missing Link in the Prediction of Birth Weight Centile. *The Journal of clinical endocrinology and metabolism* 2020; **105**(3). e-pub ahead of print 2019/12/14; doi: 10.1210/clinem/dgz248

61. Ortega-Senovilla H, van Poppel MNM, Desoye G, Herrera E. Angiopoietin-like protein 4 (ANGPTL4) is related to gestational weight gain in pregnant women with obesity. *Scientific reports* 2018; **8**(1)**:** 12428. e-pub ahead of print 2018/08/22; doi: 10.1038/s41598-018-29731-w

62. Barker G, Lim R, Georgiou HM, Lappas M. Omentin-1 is decreased in maternal plasma, placenta and adipose tissue of women with pre-existing obesity. *PLoS One* 2012; **7**(8)**:** e42943. e-pub ahead of print 2012/09/07; doi: 10.1371/journal.pone.0042943

63. Ramsay JE, Ferrell WR, Crawford L, Wallace AM, Greer IA, Sattar N. Maternal obesity is associated with dysregulation of metabolic, vascular, and inflammatory pathways. *The Journal of clinical endocrinology and metabolism* 2002; **87**(9)**:** 4231-4237. e-pub ahead of print 2002/09/06;

64. Svensson H, Wetterling L, Bosaeus M, Odén B, Odén A, Jennische E *et al.* Body fat mass and the proportion of very large adipocytes in pregnant women are associated with gestational insulin resistance. *International journal of obesity (2005)* 2016; **40**(4)**:** 646-653. e-pub ahead of print 2015/11/14; doi: 10.1038/ijo.2015.232

65. Haghiac M, Vora NL, Basu S, Johnson KL, Presley L, Bianchi DW *et al.* Increased death of adipose cells, a path to release cell-free DNA into systemic circulation of obese women. *Obesity (Silver Spring, Md.)* 2012; **20**(11)**:** 2213-2219. e-pub ahead of print 2012/07/28; doi: 10.1038/oby.2012.138

66. Alves JG, Souza ASR, Figueiroa JN, de Araújo CAL, Guimarães A, Ray JG. Visceral Adipose Tissue Depth in Early Pregnancy and Gestational Diabetes Mellitus - a Cohort Study. *Scientific reports* 2020; **10**(1)**:** 2032. e-pub ahead of print 2020/02/08; doi: 10.1038/s41598-020-59065-5

67. Bourdages M, Demers ME, Dube S, Gasse C, Girard M, Boutin A *et al.* First-Trimester Abdominal Adipose Tissue Thickness to Predict Gestational Diabetes. *J Obstet Gynaecol Can* 2018; **40**(7)**:** 883-887. e-pub ahead of print 2018/05/05; doi: 10.1016/j.jogc.2017.09.026

68. Budak MS, Kahramanoglu I, Vitale SG, Akgol S, Dilek ME, Kartal S *et al.* Maternal abdominal subcutaneous fat thickness as a simple predictor for gestational diabetes mellitus. *J Perinat Med* 2019; **47**(6)**:** 605-610. e-pub ahead of print 2019/05/30; doi: 10.1515/jpm-2018-0431

69. D'Ambrosi F, Crovetto F, Colosi E, Fabietti I, Carbone F, Tassis B *et al.* Maternal Subcutaneous and Visceral Adipose Ultrasound Thickness in Women with Gestational Diabetes Mellitus at 24-28 Weeks' Gestation. *Fetal Diagn Ther* 2018; **43**(2)**:** 143-147. e-pub ahead of print 2017/06/19; doi: 10.1159/000475988

70. D'Ambrosi F, Rossi G, Soldavini CM, Di Maso M, Carbone IF, Cetera GE *et al.* Ultrasound assessment of maternal adipose tissue during 1st trimester screening for aneuploidies and risk of developing gestational diabetes. *Acta Obstet Gynecol Scand* 2020; **99**(5)**:** 644-650. e-pub ahead of print 2020/01/04; doi: 10.1111/aogs.13800

71. De Souza LR, Berger H, Retnakaran R, Maguire JL, Nathens AB, Connelly PW *et al.* First-Trimester Maternal Abdominal Adiposity Predicts Dysglycemia and Gestational Diabetes Mellitus in Midpregnancy. *Diabetes care* 2016; **39**(1)**:** 61-64. e-pub ahead of print 2015/11/04; doi: 10.2337/dc15-2027

72. De Souza LR, Berger H, Retnakaran R, Vlachou PA, Maguire JL, Nathens AB *et al.* Hepatic fat and abdominal adiposity in early pregnancy together predict impaired glucose homeostasis in mid-pregnancy. *Nutrition & diabetes* 2016; **6**(9)**:** e229. e-pub ahead of print 2016/09/20; doi: 10.1038/nutd.2016.39

73. Gur EB, Ince O, Turan GA, Karadeniz M, Tatar S, Celik E *et al.* Ultrasonographic visceral fat thickness in the first trimester can predict metabolic syndrome and gestational diabetes mellitus. *Endocrine* 2014; **47**(2)**:** 478-484. e-pub ahead of print 2014/01/24; doi: 10.1007/s12020-013-0154-1

74. Kansu-Celik H, Karakaya BK, Tasci Y, Hancerliogullari N, Yaman S, Ozel S *et al.* Relationship maternal subcutaneous adipose tissue thickness and development of gestational diabetes mellitus. *Interv Med Appl Sci* 2018; **10**(1)**:** 13-18. e-pub ahead of print 2018/10/27; doi: 10.1556/1646.10.2018.01

75. Kennedy NJ, Peek MJ, Quinton AE, Lanzarone V, Martin A, Benzie R *et al.* Maternal abdominal subcutaneous fat thickness as a predictor for adverse pregnancy outcome: a longitudinal cohort study. *BJOG : an international journal of obstetrics and gynaecology* 2016; **123**(2)**:** 225-232. e-pub ahead of print 2016/02/04; doi: 10.1111/1471-0528.13758

76. Nassr AA, Shazly SA, Trinidad MC, El-Nashar SA, Marroquin AM, Brost BC. Body fat index: A novel alternative to body mass index for prediction of gestational diabetes and hypertensive disorders in pregnancy. *Eur J Obstet Gynecol Reprod Biol* 2018; **228:** 243-248. e-pub ahead of print 2018/07/18; doi: 10.1016/j.ejogrb.2018.07.001

77. Rocha ADS, Bernardi JR, Matos S, Kretzer DC, Schöffel AC, Goldani MZ *et al.* Maternal visceral adipose tissue during the first half of pregnancy predicts gestational diabetes at the time of delivery - a cohort study. *PLoS One* 2020; **15**(4)**:** e0232155. e-pub ahead of print 2020/05/01; doi: 10.1371/journal.pone.0232155

78. Thaware PK, Patterson CC, Young IS, Casey C, McCance DR. Clinical utility of ultrasonography-measured visceral adipose tissue depth as a tool in early pregnancy screening for gestational diabetes: a proof-of-concept study. *Diabetic medicine : a journal of the British Diabetic Association* 2019; **36**(7)**:** 898-901. e-pub ahead of print 2019/01/24; doi: 10.1111/dme.13906

79. Yang SH, Kim C, An HS, An H, Lee JS. Prediction of Gestational Diabetes Mellitus in Pregnant Korean Women Based on Abdominal Subcutaneous Fat Thickness as Measured by Ultrasonography. *Diabetes & metabolism journal* 2017; **41**(6)**:** 486-491. e-pub ahead of print 2017/12/05; doi: 10.4093/dmj.2017.41.6.486

80. Zhang YZ, Zhou L, Tian L, Li X, Zhang G, Qin JY *et al.* A mid-pregnancy risk prediction model for gestational diabetes mellitus based on the maternal status in combination with ultrasound and serological findings. *Exp Ther Med* 2020; **20**(1)**:** 293-300. e-pub ahead of print 2020/06/17; doi: 10.3892/etm.2020.8690

81. Saif Elnasr I, Ammar H. Ultrasound markers for prediction of gestational diabetes mellitus in early pregnancy in Egyptian women: observational study. *The journal of maternal-fetal & neonatal medicine : the official journal of the European Association of Perinatal Medicine, the Federation of Asia and Oceania Perinatal Societies, the International Society of Perinatal Obstet* 2020**:** 1-7. e-pub ahead of print 2020/03/07; doi: 10.1080/14767058.2019.1678132

82. Iqbal R, Rafique G, Badruddin S, Qureshi R, Cue R, Gray-Donald K. Increased body fat percentage and physical inactivity are independent predictors of gestational diabetes mellitus in South Asian women. *Eur J Clin Nutr* 2007; **61**(6)**:** 736-742. e-pub ahead of print 2006/12/21; doi: 10.1038/sj.ejcn.1602574

83. Msollo SS, Martin HD, Mwanri AW, Petrucka P. Prevalence of hyperglycemia in pregnancy and influence of body fat on development of hyperglycemia in pregnancy among pregnant women in urban areas of Arusha region, Tanzania. *BMC Pregnancy Childbirth* 2019; **19**(1)**:** 315. e-pub ahead of print 2019/08/29; doi: 10.1186/s12884-019-2463-8

84. Balani J, Hyer SL, Shehata H, Mohareb F. Visceral fat mass as a novel risk factor for predicting gestational diabetes in obese pregnant women. *Obstet Med* 2018; **11**(3)**:** 121-125. e-pub ahead of print 2018/09/15; doi: 10.1177/1753495x17754149

85. Harville EW, Juonala M, Viikari JS, Raitakari OT. Preconception metabolic indicators predict gestational diabetes and offspring birthweight. *Gynecol Endocrinol* 2014; **30**(11)**:** 840-844. e-pub ahead of print 2014/07/10; doi: 10.3109/09513590.2014.937336

86. Madhavan A, Beena Kumari R, Sanal MG. A pilot study on the usefulness of body mass index and waist hip ratio as a predictive tool for gestational diabetes in Asian Indians. *Gynecol Endocrinol* 2008; **24**(12)**:** 701-707. e-pub ahead of print 2009/01/28; doi: 10.1080/09513590802444134

87. Yamamoto S, Douchi T, Yoshimitsu N, Nakae M, Nagata Y. Waist to hip circumference ratio as a significant predictor of preeclampsia, irrespective of overall adiposity. *J Obstet Gynaecol Res* 2001; **27**(1)**:** 27-31. e-pub ahead of print 2001/05/02; doi: 10.1111/j.1447-0756.2001.tb01211.x

88. Kuzmicki M, Telejko B, Wawrusiewicz-Kurylonek N, Nikolajuk A, Zwierz-Gugala D, Jelski W *et al.* Retinol-binding protein 4 in adipose and placental tissue of women with gestational diabetes. *Gynecol Endocrinol* 2011; **27**(12)**:** 1065-1069. e-pub ahead of print 2011/05/26; doi: 10.3109/09513590.2011.579651

89. Chu Y, Liu W, Cui Q, Feng G, Wang Y, Jiang X. Analysis of phosphatidylinositol 3-kinase activation in the adipose tissue of gestational diabetes mellitus patients and insulin resistance. *J Huazhong Univ Sci Technolog Med Sci* 2010; **30**(4)**:** 505-508. e-pub ahead of print 2010/08/18; doi: 10.1007/s11596-010-0458-9

90. Tumurbaatar B, Poole AT, Olson G, Makhlouf M, Sallam HS, Thukuntla S *et al.* Adipose Tissue Insulin Resistance in Gestational Diabetes. *Metabolic syndrome and related disorders* 2017; **15**(2)**:** 86-92. e-pub ahead of print 2017/01/13; doi: 10.1089/met.2016.0124

91. Telejko B, Kuzmicki M, Zonenberg A, Szamatowicz J, Wawrusiewicz-Kurylonek N, Nikolajuk A *et al.* Visfatin in gestational diabetes: serum level and mRNA expression in fat and placental tissue. *Diabetes Res Clin Pract* 2009; **84**(1)**:** 68-75. e-pub ahead of print 2009/02/03; doi: 10.1016/j.diabres.2008.12.017

92. Ma Y, Gao J, Yin J, Gu L, Liu X, Chen S *et al.* Identification of a Novel Function of Adipocyte Plasma Membrane-Associated Protein (APMAP) in Gestational Diabetes Mellitus by Proteomic Analysis of Omental Adipose Tissue. *J Proteome Res* 2016; **15**(2)**:** 628-637. e-pub ahead of print 2016/01/16; doi: 10.1021/acs.jproteome.5b01030

93. Lou Y, Wu C, Wu M, Xie C, Ren L. The changes of neutrophil gelatinase-associated lipocalin in plasma and its expression in adipose tissue in pregnant women with gestational diabetes. *Diabetes Res Clin Pract* 2014; **104**(1)**:** 136-142. e-pub ahead of print 2014/02/18; doi: 10.1016/j.diabres.2014.01.014
